# A Peer-Led Digital Intervention to Reduce HIV Prevention and Care Disparities Among Young Brazilian Transgender Women (The BeT Study): Protocol for an Intervention Study

**DOI:** 10.2196/44157

**Published:** 2023-02-03

**Authors:** Emilia Moreira Jalil, Erin Wilson, Laylla Monteiro, Thaylla Varggas, Isabele Moura, Thiago S Torres, Brenda Hoagland, Sandra Wagner Cardoso, Ronaldo Ismerio Moreira, Valdilea Gonçalves Veloso dos Santos, Beatriz Grinsztejn, BeT Team

**Affiliations:** 1 Evandro Chagas National Institute of Infectious Diseases Oswaldo Cruz Foundation Rio de Janeiro Brazil; 2 Center for Public Health Research, San Francisco Department of Public Health San Francisco, CA United States

**Keywords:** youth, transgender women, Brazil, HIV prevention, HIV care, peer-led digital intervention

## Abstract

**Background:**

The HIV epidemic continues to disproportionately burden marginalized populations despite the availability of effective preventive and therapeutic interventions. Transgender women are severely affected by HIV worldwide including in Brazil and other low- and middle-income countries, with evidence of increasing new infections among young people. There is an urgent need for youth-specific HIV prevention and care interventions for young transgender women in Brazil.

**Objective:**

This study aims to (1) address stigma in the Brazilian public health system and (2) reduce barriers to HIV care and prevention with systems navigation among young transgender women aged 18-24 years in Rio de Janeiro, Brazil.

**Methods:**

The *Brilhar e Transcender* (BeT) study is a status-neutral, peer-led, single-arm digital intervention study enrolling 150 young transgender women in Rio de Janeiro, Brazil. The intervention was pilot tested and refined using data from a formative phase. The BeT intervention takes place over 3 months, is delivered remotely via mobile phone and in person by peers, and comprises three components: (1) BeT sessions, (2) digital interactions, and (3) automated messages. Eligibility criteria include identifying as transgender women, being aged 18-24 years, speaking in Portuguese, and living in the Rio de Janeiro metropolitan area in Brazil. The primary outcomes are HIV incidence, pre-exposure prophylaxis uptake, linkage to HIV care, and viral suppression. Primary outcomes were assessed at baseline and quarterly for 12 months. Participants respond to interviewer-based surveys and receive tests for HIV and sexually transmitted infections.

**Results:**

The study has been approved by the Brazilian and the US local institutional review boards in accordance with all applicable regulations. Study recruitment began in February 2022 and was completed in early July 2022. Plans are to complete the follow-up assessment of study participants on July 2023, analyze the study data, and disseminate intervention results by December 2023.

**Conclusions:**

Interventions to engage a new generation of transgender women in HIV prevention and care are needed to curb the epidemic. The BeT study will evaluate a digital peer-led intervention for young transgender women in Brazil, which builds on ways young people engage in systems and uses peer-led support to empower transgender youth in self-care and health promotion. A promising evaluation of the BeT intervention may lead to the availability of this rapidly scalable status-neutral HIV intervention that can be translated throughout Brazil and other low- and middle-income countries for young transgender women at high risk of or living with HIV.

**Trial Registration:**

ClinicalTrials.gov NCT05299645; https://clinicaltrials.gov/ct2/show/NCT05299645

**International Registered Report Identifier (IRRID):**

DERR1-10.2196/44157

## Introduction

Efforts to end the HIV epidemic by 2030 will not be possible without enhanced efforts to prevent new infections among youths and young adults [1]. Despite the availability of effective HIV care and prevention interventions, reductions in HIV prevalence have stalled, in part, owing to the lack of progress in reducing new infections among youths, especially those in low- and middle-income countries (LMICs) where most new infections are acquired. Innovations in HIV interventions have also failed to adequately reach marginalized populations most affected by HIV [2]. Transgender women are one of the most severely affected populations by HIV worldwide [3]. Brazil has a concentrated HIV epidemic with a 0.4% prevalence in the general population [4] with over 900,000 people living with HIV in the country [5] and the highest prevalence among transgender women who have sex with men [6,7]. Within the population of transgender women, young transgender women are at high risk for HIV and poor HIV care outcomes. The first respondent-driven sampling-based study of HIV risk and pre-exposure prophylaxis (PrEP) awareness among Brazilian transgender women identified that 24.2% of youths aged 18-24 years were living with HIV. Young transgender women also had lower knowledge of HIV prevention strategies (including PrEP) and higher HIV risk than their older peers, with 3 times as many unrecognized infections than adults [8]. These findings are worse than those reported in prior research, suggesting that 1 in 5 young transgender women were infected with HIV before having been aged 25 years [9,10].

Global data reflect a lack of incident infection decline among youths and point to a need for early intervention to curb risk among young transgender women [11]. In a study conducted from 2018 to 2020 using the Limiting Avidity assay as part of a recent infection testing algorithm, the annualized HIV incidence rate was 7% overall and 9% among transgender women [12]. In the largest PrEP demonstration study in Latin America, which enrolled a total of 9509 people, young and transgender participants had lower odds of long-term engagement in PrEP [13].

The Brazilian Public Health System (Sistema Único de Saúde) ensures universal and equitable provision of health care to the whole population; their approach toward the HIV epidemic was human rights–based and science-driven, integrating both prevention and treatment efforts into the Brazilian universal health care system and including a fundamental role of social movements advocacy. Brazilian regulations guide public health services to use social names (ie, names that transgender people use that are different from what is on their government-issued identification) and requested pronouns for them. Despite these protections, discrimination toward transgender people in the Brazilian Public Health System is well documented. For young transgender people, discrimination exacerbates common youth-related barriers to HIV prevention and care. Previous Brazilian research identified stigma as a critical roadblock to HIV prevention and care for transgender women [14,15]. Much like research from the United States, transgender women in our Brazilian study reported experiencing antitransgender stigma in the health care system [6,16-19], and most attributed their underuse of medical care to stigma [6,14]. Transgender people are known to postponing necessary care owing to experiences of and anticipation of discrimination [20,21]. Low health care usage may be the reason underlying very low awareness of PrEP in our US-based research with young transgender women [22]. Young transgender women face the challenge of addressing medical needs related to their gender identity, which compounds typical adolescent development [9,23-25]. For youths, health literacy, systems navigation, and low risk perception are barriers to HIV testing and linkage to HIV care [26-28].

The lack of interventions among young transgender women presents a troubling outlook to the HIV epidemic for young Brazilian transgender women. Overall, most interventions with transgender women focused on those already living with HIV [29]. The few primary prevention interventions are limited to pilot studies of small group sessions that combine didactic and participatory learning, with no information gathered on HIV incidence and risk behavior. Only one intervention has been developed and has shown efficacy for increasing HIV-related preventive behavior among young transgender women [30]. No known interventions have been developed to increase HIV care outcomes among young transgender women. Our data point to an urgent need for youth-specific HIV prevention and care interventions for young transgender women in Brazil. To address limitations in availability of HIV prevention and care interventions for young transgender women, we designed the *Brilhar e Transcender* (BeT [“shine and transcend” in English]) study, which combines a social marketing campaign to address antitransgender stigma in health services toward transgender women and an adapted evidence-based, HIV status–neutral intervention that uses peer-led digital and in-person systems navigation. Our primary aims are to (1) address stigma in the Brazilian public health system, (2) intervene to overcome youth challenges with health care systems navigation, and (3) implement our intervention among young transgender women aged 18-24 years in Rio de Janeiro, Brazil.

## Methods

### Study Design

The BeT study is one of 8 sponsored research projects chosen to be a part of a Eunice Kennedy Shriver National Institute of Child Health and Human Development–funded Prevention and Treatment through a Comprehensive Care Continuum for HIV-affected Adolescents in Resource Constrained Settings (PATC^3^H) consortium [31], which conducts clinical research and evaluation of a variety of combination interventions to improve health outcomes among adolescents at risk for HIV and youths living with HIV in resource-limited settings. The BeT study is the only PATC^3^H project conducted in Latin America and among transgender youths and builds on decades of research and community engagement with transgender communities. The BeT study is a peer-led, digital-based intervention study aiming to enroll 150 young transgender women in Rio de Janeiro, Brazil.

### Eligibility Criteria

Inclusion criteria are (1) having been assigned the male sex at birth and self-identifying as *travesti*, a transgender woman, or having a gender identity different from identities typically associated with the assigned sex at birth; (2) being aged between 18 and 24 years; (3) providing consent for study participation; (4) living in Rio de Janeiro or the greater metropolitan area; and (5) being sexually active. Additional criteria for HIV-negative individuals include (1) no current PrEP use, (2) PrEP indication per Brazilian standards of care, and (3) no PrEP contraindication. For participants living with HIV, additional inclusion criteria are (1) having a confirmed HIV diagnosis, (2) not currently using antiretroviral therapy (ART) or having a detectable viral load and treatment that is not linked to HIV care.

Exclusion criteria include participation in any HIV prevention or care intervention study in the last year, refusal to undergo HIV testing, or having any health condition that, in the opinion of the investigator, precludes participation in the study or may lead to harm to the participant.

### Formative Phase

**Figure 1:**
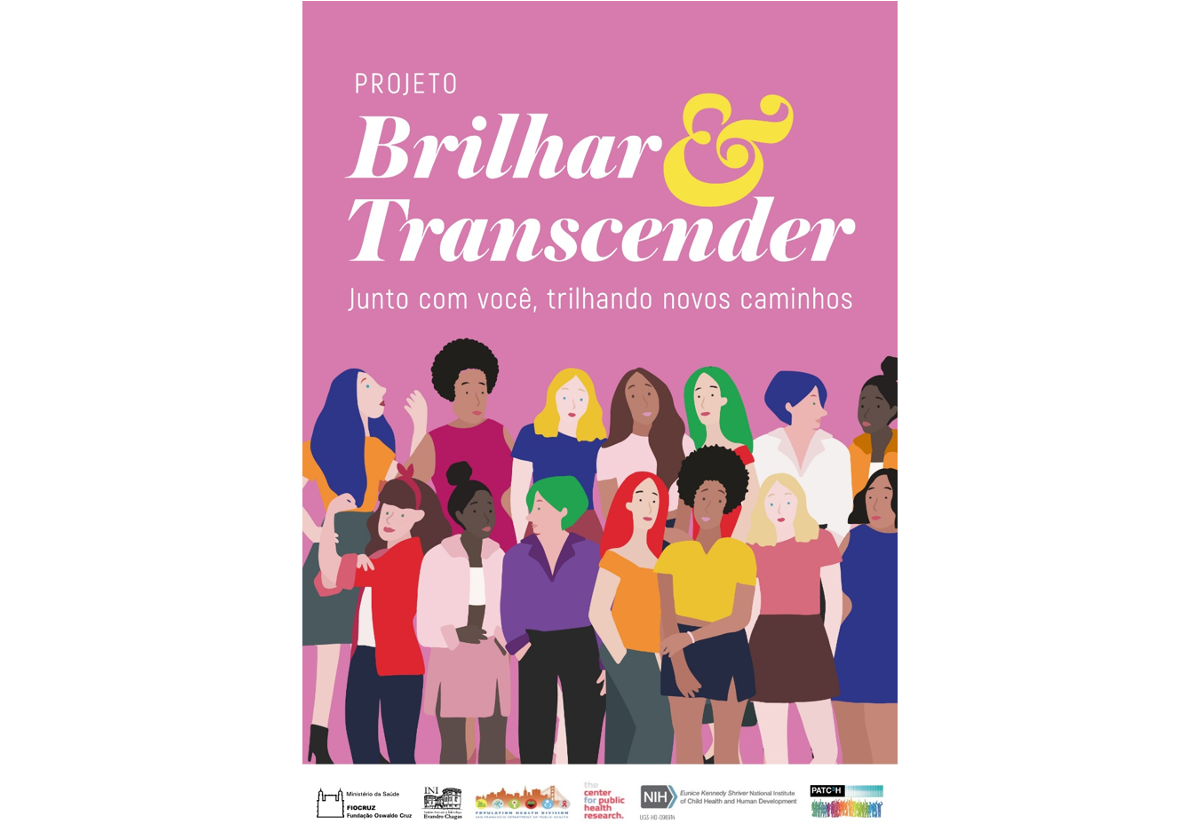
The antistigma campaign material of the *Brilhar e Transcender* study.

The study had a formative phase, which comprised developing and implementing an antistigma campaign, and adaptation and pilot testing of the system navigation intervention [32]. The campaign content included information on basic gender definitions (sex, gender identity, gender expression, and sexual orientation), regulations linked to gender and sexual orientation minorities, the medical transition process, overview of mental health and hormone therapy among transgender people, transphobia and violence against transgender people, and HIV among transgender women (Figure 1). The antistigma campaign, focused on health care settings, clinical providers, and support staff, took place at 4 health clinics serving transgender women in Rio de Janeiro, Brazil. We collected pre- and postcampaign qualitative and quantitative data with providers (N=10) to assess whether antitransgender stigma beliefs and attitudes changed among providers and support staff.

We also conducted formative qualitative interviews with 10 young transgender women (mean age 22.2 years) to inform the development of the pilot intervention. The BeT study uses an effective US Centers for Disease Control and Prevention intervention called *Anti-Retroviral Treatment and Access to Services* for system navigation and linkage [33,34]. Three components of the intervention required adaptation: (1) content of the Anti-Retroviral Treatment and Access to Services intervention session plan and asynchronous interactions with young transgender women, (2) adaptation to the cultural context of Brazil, and (3) the digital interface. Ten young transgender women were recruited through community outreach by our community engagement specialists (n=2) and through referrals from other studies at Fiocruz (n=8).

The BeT study pilot-assessed the intervention’s preliminary efficacy among 20 participants. Results from the pilot intervention demonstrated preliminary efficacy for improving HIV prevention and care behaviors among young transgender women, as all HIV-negative participants were engaged in PrEP and all but one participant living with HIV became virally suppressed [32]. After the pilot phase, the team refined the intervention, which focused on streamlining the adapted intervention manual to improve peer navigator and participant accessibility. In addition, the team added additional medical monitoring to include important biomarkers of sexual health and HIV as part of the study protocol.

### Study Procedures and Measures

Table 1 describes the study procedures. Potential participants are recruited by a community education team, through word of mouth, and via referrals from enrolled participants. Recruitment also occurs at the HIV prevention services based at our site and at an on-site mobile legal unit that offers weekly access to free legal services for transgender people.

Participants have 5 in-person encounters at the National Institute of Infectious Diseases Evandro Chagas, Fiocruz, including a screening and enrollment visit followed by 4 quarterly visits (at weeks 12, 24, 36, and 48). The BeT intervention takes place between the enrollment visit and the 12-week visit and is completed within that time frame. Trained staff conduct interviewer-administered surveys to participants at all study visits.

At baseline, all participants respond to a screening questionnaire and demographic information, after signing the written informed consent form. Baseline and quarterly survey content build upon questionnaires previously developed and tested with transgender women in Brazil and in the United States, as described in Table 2. During follow-up visits, we will collect information on study outcomes: HIV incidence, PrEP uptake and adherence among HIV-negative participants, and linkage to HIV care and viral load suppression among those living with HIV. In addition, we will also collect data on implementation science outcomes: (1) maintenance (pre- and poststudy) for study coordination, feasibility (poststudy) for peer health navigators, and feasibility, acceptability, and appropriateness (poststudy) for study participants.

**Table 1:** Measures of the *Brilhar e Transcender* study.

Measures		Time frame	Source
Demographics		Baseline	Grinsztejn et al [6]
Transitioning process		Baseline and quarterly	Grinsztejn et al [6]
Food insecurity		Baseline and quarterly	Adapted from the Food and Agriculture Organization of the United Nations [35]
**Substance use**			
	Alcohol Use Disorders Identification Test for alcohol consumption (3 items)	Baseline and quarterly	Bush et al [36]
	Alcohol, Smoking and Substance Involvement Screening Test	Baseline and quarterly	World Health Organization [37]
Stigma and discrimination (including transgender-specific health care discrimination)		Baseline and quarterly	Grinsztejn et al [6], Costa et al [15], and Scandurra et al [38]
Violence		Baseline and quarterly	Grinsztejn et al [6]
Overall health issues		Baseline and quarterly	Grinsztejn et al [6]
Medical mistrust (12-item Group-Based Medical Mistrust Scale)		Baseline and quarterly	Adapted from Thompson et al [39]
Medical avoidance		Baseline and quarterly	Adapted from James et al [40]
HIV knowledge and attitudes (10-item HIV Knowledge Questionnaire adapted to 8 items, HIV testing and counseling attitudes and knowledge)		Baseline and quarterly	Oglesby and Alemagno [41] and Kalichman and Simbayi [42]
Self-efficacy			Adapted from Zhao et al [43]
HIV testing, prevention, and care		Baseline and quarterly	Grinsztejn et al [6]
HIV perceived risk index		Baseline and quarterly	Lauby et al [44]
Testing and diagnosis of sexually transmitted infections		Baseline and quarterly	Grinsztejn et al [6]
Adherence (adapted to antiretroviral therapy and pre-exposure prophylaxis)		Baseline and quarterly	Adapted from Wilson et al [45]
Overall mental health assessment and suicide		Baseline and quarterly	Grinsztejn et al [6] and Jalil et al [46]
The Patient Health Questionnaire for Depression and Anxiety scale		Baseline and quarterly	Kroenke et al [47]
The Primary Care Posttraumatic Stress Disorder scale		Baseline and quarterly	Prins et al [48]
Social support (Multidimensional Scale of Perceived Social Support)		Baseline and quarterly	Zimet et al [49]
Resilience (2-item Connor-Davidson Resilience scale)		Baseline and quarterly	Connor and Davidson [50]
Brazilian political context		Baseline and quarterly	The authors of this study
Sexual behavior		Baseline and quarterly	Adapted from Rocha et al [51]

**Table 2:** Schedule of events of the *Brilhar e Transcender* study.

			Baseline		Week 12		Week 24		Week 36		Week 48 or early termination^a^		HIV seroconversion visit
Eligibility			✓										
**Measures**													
	Behavioral, partnership, network, and structural measures	✓		✓		✓		✓		✓		✓	
	Adverse events			✓		✓		✓		✓			
	Social harm			✓		✓		✓		✓			
	Implementation science: participants									✓			
	Implementation science: coordination	✓								✓			
	Implementation science: peer navigators									✓			
**Laboratory procedures**													
	HIV rapid test	✓		✓^b^		✓^b^		✓^b^		✓^b^		✓	
	CD4^+^ and CD8^+^ cell counts	✓^c^				✓^c^				✓^d^		✓	
	HIV RNA viral load	✓^c,d^		✓^d^		✓^c,d^		✓^d^		✓^c,d^		✓	
	HIV recency testing	✓^c^										✓	
	HIV genotyping	✓^c^										✓	
	Treponemal syphilis rapid test	✓		✓		✓		✓		✓			
	Nontreponemal syphilis (venereal disease research laboratory) testing	✓^e^		✓^e^		✓^e^		✓^e^		✓^e^			
	Hepatitis B rapid test	✓								✓^f^			
	Hepatitis B serology	✓^g^											
	Hepatitis C rapid test	✓								✓^h^			
	Anti–hepatitis C virus	✓^i^								✓^i^			
	Hepatitis C viral load	✓^j^								✓^j^			
	Rectal swab (*Chlamydia trachomatis* and *Neisseria gonorrhoeae*)	✓								✓			
	Dried blood spot (pre-exposure prophylaxis adherence assessment)	✓^k^		✓^k^		✓^k^		✓^k^		✓^k^		✓^k^	
Hormone dispensation			✓		✓		✓		✓		✓		✓
Medical or psychiatric visit			✓^l^		✓^l^		✓^l^		✓^l^		✓^l^		

^a^Withdrawn or discontinued participants before the final visit.

^b^Only for participants with a negative HIV test in the previous visit.

^c^For all participants living with HIV.

^d^Only for HIV-negative participants with recent HIV exposition according to the service guidelines.

^e^Only for those with a positive treponemal test result.

^f^Only for a negative hepatitis B rapid test result.

^g^Only for a positive hepatitis B rapid test result.

^h^Only for those with a negative hepatitis C rapid test result.

^i^Only for those with a positive hepatitis C rapid test result.

^j^Only for those with positive findings on an anti–hepatitis C virus antibody test.

^k^Only for participants receiving pre-exposure prophylaxis, except those initiating pre-exposure prophylaxis at the current visit.

^l^If clinically indicated.

### The BeT Intervention

**Figure 2:**
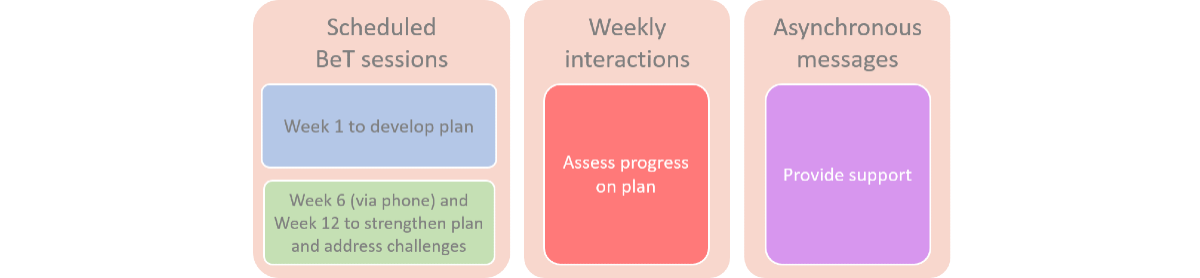
Intervention components of the Brilhar e Transcender study.

The 3-month BeT intervention comprises three components: (1) BeT sessions, (2) digital interactions, and (3) automatic messages (Figure 2). During the first BeT session, the participant works with a peer navigator to build a case management plan aimed at meeting HIV prevention and care goals, and getting other needs met to promote health and wellness (BeT plan). BeT sessions 1 and 3 are conducted in person at enrollment and week 12 visits, respectively. BeT session 2 is conducted remotely via a mobile phone. Participants select a preferred app (eg, WhatsApp or Facebook messenger) for communicating asynchronously with the peer navigator during BeT session 2. Asynchronous digital interactions with peer navigators occurs at least weekly for 3 months and can be more frequent based on participant needs. Digital interactions were not restricted to study participation or HIV prevention or care and can include social support, food basket provisions, and guidance on employment, self-care, and other supports. Finally, participants received weekly automatic messages with health information on HIV testing, prevention, and care.

### Statistical Analysis

Statistical analysis will assess recruitment, baseline, and follow-up data and will describe the rapidity of enrollment of young transgender women, their demographic and risk characteristics, and participation as the percent intervention uptake (ie, the number of participants in the BeT intervention divided by the total number of individuals who were assessed for participation and were preliminarily eligible for the study). We will describe PrEP uptake and adherence and HIV care service linkage at intervention initiation and completion. For the primary endpoint, we will compare PrEP uptake and adherence and HIV care linkage between baseline and follow-up data (ie, before and after the intervention). Primary analyses will test the hypothesis that the odds of PrEP uptake and adherence and HIV care linkage will be higher post intervention relative to baseline data. We anticipate behavior change within 3 instead of 6 months because of our ability to more frequently interact with participants (3 months). In fact, we may achieve a greater dose response and intervention effect in a shorter amount of time because of the possibility for a higher number of encounters. At minimum, participants will engage with their digital navigator 4 to 5 times a week in the form of short digital encounters, and over the course of the intervention, participants will have 48 to 60 short digital encounters in total. We will use a pooled logistic model to estimate treatment effects on time to PrEP uptake and adherence and HIV care linkage.

We will also assess outcomes comparing data from the external comparison group to the BeT intervention group. First, we will conduct sensitivity analyses to compare the demographics of our intervention group to those of the external comparison group. In the analysis, we will focus on comparisons between the BeT intervention group and the external comparison group on measures of PrEP and ART adherence and viral suppression. Primary analyses will involve intention-to-treat analysis without considering adherence to the BeT intervention. This analysis will be a comparison of initiators of PrEP and ART, and we shall adjust for baseline confounders related to age or race. We have chosen to compare initiators of PrEP or ART and not assess PrEP uptake because a comparison of initiators parallels intention-to-treat analysis in which assignment and initiation of the treatment strategies occur together at baseline, regardless of whether individuals continue on the strategies after baseline [52]. We will combine outcome estimates with the cost estimates of the intervention component to calculate cost per participant and roughly estimate costs per HIV infection averted or poor health outcomes based on cohort data being collected in Brazil on HIV incidence and care outcomes.

### Antistigma Campaign Evaluation

Data from the baseline and follow-up study surveys will be used to assess whether there were improvements among young transgender women in (1) feeling of safety in health clinics; (2) perceptions of provider, staff, and other patient attitudes toward transgender women; (3) anticipated stigma; (4) medical mistrust; (5) health care participation; and (6) avoidance of health care. We will use repeated measures ANOVA to compare changes in mean stigma scores over the course of time in participating in the intervention (ie, before and after the intervention). In intent-to-treat analyses, we will use generalized estimating equations (GEE) Poisson or negative binomial models with robust SEs to compute the ratio of the single arm intervention rates of change in the mean value of stigma over the study period. GEE methods produce estimates that account for within-subject correlation of stigma scores across time [53]. GEE methods will also be used to assess whether changes in stigma are associated with changes in PrEP uptake and adherence and HIV care linkage over time.

### Sample Size and Power Calculation

We propose to enroll 150 participants and have estimated 80% power to detect effects for our primary outcomes using formulas for longitudinal study designs with attrition. These estimates account for an expected attrition of 15% of the sample by 12 months, as well as adjustment for time-dependent covariates in longitudinal analysis. We will also have 80% power to detect correlations of 0.23 of continuous baseline covariates with baseline responses and changes in study responses from baseline to 6 months.

In the analysis of changes, we will have 80% power in 2-sided tests with a type I error rate of 5% to detect changes from baseline to 12 months of 0.41 to 0.53 SDs in continuous responses, depending on within-subject correlation of the response, and increases of 10-15 percentage points in the frequency of binary responses, depending on baseline frequency as well as within-subject correlation. Thus, this study is powered to detect small to medium effect sizes that are consistent with or smaller than others reported in the literature.

### Missing Data

Missing items at any visit should be minimal owing to the electronic data collection protocol described above. However, some loss to follow-up is inevitable. To assess sensitivity to dropout, multiple imputation will be used, under both the standard conditionally missing-at-random assumption, as well as plausible missing not-at-random scenarios. GEE methods can also be weighted to handle missing data.

### Laboratory Assessments and Clinical Approach

HIV rapid tests will be performed at baseline for all participants and quarterly for HIV-negative participants. Participants reporting recent anal condomless sex (<30 days) with a negative HIV rapid test will be screened for acute HIV infection through HIV RNA viral load testing. The Limiting Antigen Avidity assay will be used to identify recent HIV seroconversions and may further support the calculation of HIV incidence by providing the timing of seroconversion [54]. Syphilis will be screened using treponemal testing, followed by venereal disease laboratory testing if the former test yields a positive result. Hepatitis B and C will be first screened using rapid tests followed by serology assessments, if needed. All participants will collect rectal swabs at baseline and the 48-week visit for the diagnosis of *Chlamydia trachomatis* and *Neisseria gonorrhea* infections. Sexually transmitted infections will be treated in accordance with Brazilian guidelines [55]. Participants who screened positive for hepatitis B and C will undergo complete the diagnosis algorithm (ie, hepatitis C viral load) [56] and will be referred to treatment. Participants with negative hepatitis B test results who have not been vaccinated will be referred for hepatitis B vaccination. PrEP adherence will be evaluated on the basis of tenofovir diphosphate concentrations in dried-blood spots.

Medical and psychiatric evaluation could occur as needed. Study referrals may include endocrinology, mental health evaluation, and proctology, among others. Participants may also access feminizing hormones on site.

### Ethical Considerations

The BeT intervention study was reviewed and approved by the institutional review board of the National Institute of Infectious Diseases, Fiocruz (#CAAE 05018818.0.0000.5262) and the University of California, San Francisco Committee on Human Research (#18-26770) in accordance with all applicable regulations. All participants were asked to sign an informed consent form prior to any study procedure.

## Results

Between the study pilot and the study intervention, the COVID-19 pandemic emerged, which caused a substantial impact on the implementation of the study. Study recruitment began in February 2022, initially slowly as Brazil was still facing high numbers of COVID-19 cases and deaths. Nevertheless, we were able to complete study enrollment by early July 2022. To achieve that, we combined prior experience of both the US and the Brazilian teams with transgender communities to new recruitment strategies. The team uses dynamic and multifaceted approaches to community engagement strategies to enhance existing strategies and implement new strategies using field and digital media initiatives for recruitment. The team also encouraged participants to bring other individuals and gave them gifts or reimbursement (eg, peer referrals).

Recruiting and retaining transgender women who are young, early in their gender development, marginalized, and less connected within community poses significant challenges to engagement in the intervention and retention. Therefore, the study team currently has a major focus on retention. Strong supervised peer navigation is important to reach, engage, and retain young transgender women. Regular and meaningful engagement with the community is essential to maintain these connections. Furthermore, we have been providing cell phone credits to the participants retained in the intervention. In addition, we also give participants small gifts, such as study bags, sponge bags, and daily planners, among others, if they achieve some goals (eg, attending study visits in the study window). In addition, we will also conduct raffles of electronic items (such as portable cell phone chargers and earphones) for participants completing the intervention and study visits. Peer navigators also maintain close connections with participants, supporting them in their in-person and remote interactions. Future perspectives are to complete follow-up with study participants by July 2023 and to analyze study data and disseminate intervention results December 2023.

## Discussion

The BeT study is a pivotal study that will evaluate a digital peer-led intervention for young transgender women in a LMIC such as Brazil. We anticipate that the BeT intervention will successfully improve HIV prevention and care outcomes among young transgender women. A linkage to prevention intervention is particularly important once oral PrEP is a public health policy in Brazil. Oral PrEP requires strong care linkage, engagement, and retention to reap the prevention benefits. Although oral PrEP has been implemented since 2017 in Brazil, only a small percentage of PrEP users are transgender women [57]. Ancillary services and systems navigation to such services related to PrEP are essential supports to ensure success on PrEP. Among young cis women in Africa, PrEP uptake was facilitated when offered as part of an integrated sexual reproductive health service [58]. Based on the findings of the formative phase [32], BeT intervention will be the first intervention tested to meet the needs of the transgender women population in LMICs and may be implemented in resource-constrained settings. We also expect stigma to be reduced over time as participants are engaged in a gender-affirming transgender-centered clinic where an antistigma campaign has been conducted.

The study has some limitations. First, the BeT intervention will not be tested in a randomized clinical trial. Owing to the heightened targeting of transgender people, randomizing BeT participants seemed unethical and impractical in our context. To address this issue, we will assess intervention effectiveness by monitoring changes in intervention outcomes before and after the intervention and comparing outcomes from our cohort to those in an external comparison group. Furthermore, our study will have a convenience sample and our results may not be applicable to all young transgender women. Our study also has some strengths. The study team has extensive experience in conducting transgender-related research. The Brazilian site has a successful history of engaging transgender women in relevant clinical studies owing to historical community engagement efforts that solidified the relationship with the local transgender population.

In the current local adverse political environment, where transphobia is rampant, the implementation of a transgender-specific study is an opportunity to engage a new generation of transgender women in health care. Moreover, peer-led support may empower transgender youth toward their self-care and health promotion. The BeT intervention may be rapidly brought to scale and translated to a relevant, replicable, sustainable, and evidence-based combination HIV prevention intervention for young transgender women in LMICs.

## Appendix

Multimedia Appendix 1 External peer review from the National Institute of Child Health and Human Development Special Emphasis Panel-Eunice Kennedy Shriver National Institute Of Child Health and Human Development (National Institutes of Health, USA).

## Abbreviations

ART: antiretroviral therapy
BeT: Brilhar e Transcender
GEE: generalized estimating equations
LMIC: low- and middle-income country
PATC3H: Prevention and Treatment through a Comprehensive Care Continuum for HIV-affected Adolescents in Resource Constrained Settings
PrEP: pre-exposure prophylaxis

## Data Availability

We and our institutional review board have ethical concerns with releasing our study data publicly owing to the small numbers of transgender persons, the high likelihood that sharing the primary data may expose individual participants, and the possible harm that might befall participants if exposed.
