# Supplementary material for: A Peer-Led Digital Intervention to Reduce HIV Prevention and Care Disparities Among Young Brazilian Transgender Women (The BeT Study): Protocol for an Intervention Study
Source: JMIR Res Protoc. 2023 Feb 3;12:e44157. doi: 10.2196/44157 (PMC9938440; doi:10.2196/44157)
Supplement: Multimedia Appendix 1 [file resprot_v12i1e44157_app1.pdf]

SUMMARY STATEMENT
(Privileged Communication)

PROGRAM CONTACT:
Bill Kapogiannis
(301)402-0698
kapogiannisb@mail.nih.gov

Release Date: 05/04/2018
Revised Date:

Application Number: 1 UG3 HD096914-01

Principal Investigators (Listed Alphabetically):

GRINSZTEJN, BEATRIZ
MEEK, ERIN (Contact)

Applicant Organization: PUBLIC HEALTH FOUNDATION ENTERPRISES

Review Group: ZHD1 DSR-N (52)
National Institute of Child Health and Human Development Special Emphasis Panel
AIDS - EXP. REV.

Meeting Date: 04/09/2018
Council: MAY 2018
Requested Start: 06/01/2018
RFA/PA: HD18-032
PCC: MPIDB-WK
Dual IC(s): MD

Project Title: The BeT intervention to reduce HIV prevention and care disparities among young transwomen in Rio De Janeiro
SRG Action: Impact Score:32
Next Steps: Visit https://grants.nih.gov/grants/next_steps.htm
Human Subjects: 10-No human subjects involved
Animal Subjects: 10-No live vertebrate animals involved for competing appl.

Project Year	Direct Costs Requested	Estimated Total Cost
1	221,267	258,941
2	272,123	318,456
3	254,123	297,392
4	226,322	264,857
5	242,584	283,888
TOTAL	1,216,419	1,423,534

ADMINISTRATIVE BUDGET NOTE: The budget shown is the requested budget and has not been adjusted to reflect any recommendations made by reviewers. If an award is planned, the costs will be calculated by Institute grants management staff based on the recommendations outlined below in the COMMITTEE BUDGET RECOMMENDATIONS section.

1UG3HD096914-01 Meek, Erin

RESUME AND SUMMARY OF DISCUSSION: This research application proposes to collect pilot efficacy data for an anti-trans stigma reduction campaign for young transwomen ages 18-24 years old in the Brazilian public health system in addition to a digital intervention to prevent and address HIV. This work has the potential to decrease HIV among transwomen in low and middle-income countries. The investigative team is strong with complementary expertise and experience, has two decades of experience, has worked in Brazil in the past, and a strong publication record. The team has a history of collaboration and has experience with PrEP, transgender communities, and competency in multi-center research trials. The formative research component examines the specific needs of transwomen, and this is novel as it builds on prior research to foster recruitment. The research approach is well-reasoned methodology; scientifically justifies inclusion/exclusion criteria; has a strong recruitment process; uses existing linkage and navigation technology; has appropriate go/no-go criteria; includes survey data; appropriately manages participant risk; and has preliminary pilot data demonstrating the stigma associated with transwomen and how clinics are not youth friendly. The approach also supports study rigor with the RCT and uses survey data to analyze mechanisms and moderators. The research environment is strong and has extensive resources to adequately conduct the proposed work. Application weaknesses include: a missed opportunity to add an adolescent expert to the research team; inadequately addresses participant protections, failing to address HIV prevention and care continuum as a whole but only in terms of PreP; missed opportunity to evaluate individual behavior related to alcohol use and abuse; lost opportunity to gain valuable anti-stigma information from face to face encounters; and an inadequate explanation of "digital encounters." Overall, reviewer enthusiasm was medium to high impact with the application scoring in the range of very good to outstanding.

DESCRIPTION (provided by applicant): Our recent research discovered that almost 25% of a population-based sample of young transwomen in Brazil tested positive for HIV. This finding is consistent with research demonstrating that transwomen are the population most severely affected by HIV worldwide and face important barriers to accessing effective prevention and care programs. There is no HIV prevention intervention for young transwomen with evidence of efficacy outside the US. We propose a two-phase combination prevention intervention to address the main barriers to HIV prevention and care found in our research in this resource constrained setting. Stigma prevents young transwomen from accessing HIV prevention and care services, despite the fact that such services are freely available to all Brazilians in the public health system care system (i.e., SUS). Young people's HIV testing levels and care access is uneven. Data from our study show that almost no young transwomen regularly got tested for HIV, and given low testing levels, it is unlikely youth are accessing HIV care. In response to NICHD RFA-HD-18-032, we propose the Brilho e Transcender (BeT, or Shine and Transcend) intervention for young transwomen ages 18-24 years old in Brazil. Our proposed intervention uses expertise gleaned from a US based cohort study of young transwomen and knowledge from our cross-sectional transwomen HIV risk study in Brazil. Our study is comprised of two phases to address stigma in the public health system, intervene to overcome youth challenges with health care systems navigation and to scale the intervention widely if proven efficacious. During the 2-year UG3 phase, we propose to develop, implement and measure a highly visible, community-informed social marketing campaign to reduce anti-trans stigma in four SUS clinics currently implementing national PrEP access efforts that are also part of ImPrEP, which is a 3-country PrEP implementation project focused on transgender individuals and MSM. During the UG3 phase, we will also collect formative data to adapt our ARTAS-based system navigation intervention to the cultural context and HIV prevention and care needs of young transwomen in Brazil. After adaptation, we will conduct a small pilot intervention with 20 youth participants to determine preliminary efficacy and demonstrate our ability to recruit youth in this population. Preliminary data gathered from the RCT in the UG3 phase will be used to refine the intervention and justify movement to the UH3 phase and larger RCT to test efficacy of the intervention. During the UH3 phase, we will conduct a RCT with 150 young transwomen

randomized to the BeT intervention or control. The intervention will be a digital systems navigation intervention utilizing peers to address youth-specific barriers to HIV prevention and care- namely, risk perception, system navigation skills and health literacy. The intervention will be intensively implemented over three months with follow-up to twelve months. The control arm will receive unidirectional educational text messages for the same period. We will test whether intervention arm participants have an increase in HIV testing frequency, PrEP uptake and HIV care linkage compared to participants in the control arm. A key innovation of our intervention is that it will be delivered digitally by trained transwomen peers. Interventions that require participants to interact on a particular schedule and in-person present a number of problems for young transwomen who face substantial competing needs and barriers to participation. Our intervention is private, can occur on a schedule convenient to participants, and does not require in-person interaction. The latter advantage is particularly salient for young transwomen who risk threats and abuse in public, especially on transportation. The system-navigation intervention promises multiple advantages over existing interventions that require in-person attendance, including more frequent engagement and lower cost of a digital intervention, alignment with the ways young transwomen socialize and interact, and in addressing safety challenges trans people face with participation in placed-based interventions. With proof of efficacy, the intervention can be rapidly brought to scale as a replicable, sustainable, evidence-based intervention for young transwomen in collaboration with the ImPrEP study and the Brazilian Ministry of Health efforts to increase prevention and care access for transwomen, including youth.

PUBLIC HEALTH RELEVANCE: There is not one study with proven efficacy for reducing HIV risk among young transwomen and little research with transwomen in low and middle-income countries. We propose to develop and implement a combination intervention aimed at increasing HIV prevention and care health seeking behaviors among young transwomen in Brazil. This study will provide pilot efficacy data for an anti-trans stigma reduction campaign in the Brazilian public health system along with a digital intervention to meet the needs of this youth key population in dire need of interventions to prevent and address HIV.

CRITIQUE 1

Significance: 1
Investigator(s): 1
Innovation: 1
Approach: 2
Environment: 1

Overall Impact: The team proposes to develop an intervention for young transwomen ages 18-24 years old in Brazil entitled, “Brilho e Transcender (BeT, or Shine and Transcend).” There are two phases of this study – the first phase, the 2-year UG3 will develop, implement and measure a highly visible, community-informed **social marketing campaign** to reduce anti-trans stigma in four SUS clinics currently implementing national PrEP access efforts that are also part of ImPrEP, which is a 3-country PrEP implementation project focused on transgender individuals and MSM. The second phase, will collect formative data to adapt an ARTAS-based system navigation intervention to the cultural context and HIV prevention and care needs of young transwomen in Brazil. This adaption will be followed by a small and subsequently larger pilot intervention to test the efficacy of the intervention. The intervention will be a digital systems navigation intervention utilizing peers to address youth-specific barriers to HIV prevention and care- namely, risk perception, system navigation skills and health literacy.

An application does not need to be strong in all categories to be judged likely to have major scientific impact. For example, a project that by its nature is not innovative may be essential to advance a field.

1. Significance:

Strengths

- Brazil is a middle-income country with high rates of HIV infection. Transgender women is a key population with alarming HIV rates (30% living with HIV). The investigative team have demonstrated (in prior work) that 1 in 5 adolescents and young transwomen age 18-24 are living with HIV.
- Few prevention interventions specifically focus on transwomen specifically. In fact, prior interventions have not demonstrated efficacy in this age range.
- The uniqueness of this proposal is its focus on stigma in the health care setting. The proposal seeks to address stigma/barriers to care using a multi-level system approach and leverages innovative technology with digital navigators to do so. If successful, these findings can be disseminated to other Latin communities. The Brazilian team also has a 15-site PrEP implementation demonstration project for men who have sex with men (MSM) and transgender in individuals at high risk for HIV infection in Brazil, Mexico and Peru called ImPrEP allowing for on-going feedback and dissemination between settings.

Weaknesses

- Brazil is a higher middle-income country and may not have the same income disparities as seen in other settings.

2. Investigator(s):

Strengths

- History of successfully working with transwomen interventions (including recruitment and interventions).

Weaknesses

- No adolescent-specific researchers are included. Given the developmental nature of the proposal, would include this area of expertise.

3. Innovation:

Strengths

- This is a really innovative proposal. It focuses on transgender women living in Brazil – a population with very high rates of HIV infection. If found to be effective, it can be disseminated to other Latin American sites.
- The proposed BeT intervention will utilize an effective US Centers for Disease Control and Prevention (CDC) intervention - Anti-Retroviral Treatment and Access to Services (ARTAS), an individual-level, multi-session, time-limited intervention with the goal of linking recently diagnosed persons with HIV to medical care soon after receiving their positive test result. It uses a strengths-based case management approach. It is rooted in Social Cognitive Theory (particularly self-efficacy) and encourages the client to identify and use personal strengths; create goals for himself/herself; and establish an effective, working relationship with the Linkage Coordinator (LC) to successfully link and engage into care.
- The use of a digitally-based peer navigator is innovative and will further boost retention and engagement in the intervention. Additionally, implementation of the ARTAS potentially will

address stigma, threats and abuse experienced in public places (which can create barriers to care). Its use of social media, e-mail, and texts additionally makes it more relevant for young transwomen.

- The proposal seeks to focus on transwomen 18-24 years old. The investigative team addresses this as a potential limitation given the requirement of written consent from both parents and/or guardians.

Weaknesses

- Phase 1 will be centered around adapting the ARTAS for the phase 2 implementation. The ARTAS intervention is specific to treatment linkage/access and not preventive linkage access. The investigative team has prior experience/preliminary work around working with transwomen, using SMS technology, etc, but not specifically around adaptation of this approach for prevention.
- The team has not considered the specific developmental needs and how such an approach may apply for transwomen adolescents who are very pre-contemplative around prevention and very concrete. The authors plan to conduct a focus group with younger (18-20) and older (22-24) transwomen to capture differences in risk perception, and to offer differing insights on multiple minority status, which may equate to varying perspectives on potential intervention components and feasibility of addressing specific protective factors (e.g., parental closeness). This should not only be limited to a focus group but should be considered regarding recruitment of the sample and implementation of the intervention.
- The RFA focuses on characterizing and improving upon the critical milestones of the HIV prevention continuum to reduce HIV infections among uninfected youth at high risk for HIV infection and to improve the proportion of youth living with HIV who successfully achieve each milestone on the HIV care continuum. The proposal centers only on PrEP and linkage to HIV care but not the entire HIV prevention and care continuum. The ARTAS is specific to persons living with HIV and has been used in transwomen living with HIV. The application would be strengthened to focus on both transwomen at risk for and living with HIV.
- It would be useful to include questions about medical and or surgical transition and the impact of acceptance in engaging in care. The statement from the team that “young transwomen (in the U.S.) are often focused on reconciling their gender identity during adolescence, and will often delay a social and medical transition until 18 years old, and/or when they have moved out of their parents’ house, “may not apply to Brazilian women and should be examined scientifically. By focusing on this the authors gain the ability to examine developmental stages that are key in gender development and critical transition points that directly may impact engagement in care.
- While the proposal acknowledges the age limitation of the proposal given the requirement of written consent from both parents and/or guardians, the proposal would be strengthened if the team would examine how this acts as a barrier or facilitator into care and interventions. The intervention could be modified to address this key issue. The findings would significantly impact care in other settings.

4. Approach:

Strengths

- There are two phases in this approach. The first phase will begin with a two-year marketing campaign to reduce anti-trans stigma in four ImPrEP study SUS clinics in Rio de Janeiro, Brazil. This phase will also include formative interviews and a small pilot with 20 youth. In phase two, the proposal will recruit 150 participants to participate in a BeT intervention that uses digital systems navigation intervention that leverages peers to address youth-specific barriers to HIV

prevention and care- namely, risk perception, system navigation skills and health literacy. By using this approach, the application is responsive to the RFA to employ a variety of innovative combination interventions aimed at the individual, family, clinic, community, structural and education and health systems levels.

- The proposal also leverages established community ties to implement its intervention. The campaign proposed in phase 1 will use positive opinion leaders to develop campaign materials created for and developed specifically to address stigma experienced by trans youth.
- The design team has years of experience in website development, logos, branding, and print design, and has developed highly effective, eye-catching campaigns for recruitment to various HIV prevention studies (including the Stay Study (www.staystudy.org) campaign that will be modified for this project).
- The project uses tiered incentives to motivate continued participation. The project team has a history of high retention. Face-to-face interviews will be conducted at 3, 6, 9, 12.

Weaknesses

- There is no contingency identified in this proposal if the preliminary phase is not successful. The investigators state that preliminary data gathered from the RCT in the UG3 phase will be used to refine the intervention and justify movement to the UH3 phase and larger RCT to test efficacy of the intervention. What if the pilot is unsuccessful? What will be the plan for the UH3 phase? An alternative plan would strengthen the proposal.
- Has the raffle been used in other adolescent/young adult specific projects led by the team? Additional justification is required with this approach.
- Will the navigators be same-age peers? What protections will be put into place to insure the safety of adolescents and young adults 18-24 working with potentially older peers?
- The authors should account for clustering at SUS clinics that might impact the outcome of the proposal. The sample size did not account for potential clustering within person and across clinic sites.
- Please specify the security measures taken to insure confidential protections are in place for participants who may be living in their parent's home and whose participation in the study might disclose their identity.
- Besides linking to services, there is very little focused on addressing individual behavioral and/or community level combination interventions which screen for alcohol problems, and address the pattern and severity of use and early initiation of alcohol, to reduce HIV incidence among at risk youth and/or improve disease course among youth living with HIV.

5. Environment:

Strengths

- Long-standing history working in Brazil. The team is a part of ImPrEP, which is a 3-country PrEP implementation project focused on transgender individuals and MSM. Interventions have occurred both within and outside of Brazil.

Weaknesses

- No concerns.

— Responses for Protections for Minor Participants, Human Subjects, Vertebrate Animals, and

— Biohazards:

— A response for Inclusion of Women, Minorities and Children **is required from reviewers** for applications proposing Human Subjects Research

Protections for Minor Participants:

- The proposal has focused on adolescents and young adults 18 years and older due to concerns about the ability to enroll persons under the age of majority. More details are needed to insure adolescents above the age of majority are protected, especially given the use of digital technology and potential disclosure of confidential information with navigators.

Protections for Human Subjects: Acceptable Risks/Adequate Protections

Data and Safety Monitoring Plan (Applicable for Clinical Trials Only): Acceptable plan.

- o While not a clinical trial, would recommend a DSMB for this study to insure protections are in-place for adolescents and young adults. Especially when proceeding into phase 2.

Inclusion of Women, Minorities and Children:

- Sex/Gender: Distribution justified scientifically
- Race/Ethnicity: Distribution justified scientifically
- For NIH-Defined Phase III trials, Plans for valid design and analysis: Not applicable
- Inclusion/Exclusion of Children under 18: Exclusion of children under 18 justified.

Vertebrate Animals:

Biohazards:

Applications from Foreign Organizations:

Select Agent Research:

Resource Sharing Plans:
(1) Data Sharing Plan; (2) Sharing Model Organisms; and (3) Genomic Data Sharing Plan (GDS).

Authentication of Key Biological and/or Chemical Resources:

Budget and Period of Support:

Recommended budget modifications or possible overlap identified:

Additional Comments to Applicant (Optional):

CRITIQUE 2

Significance: 2
Investigator(s): 1
Innovation: 3
Approach: 3
Environment: 2

Overall Impact: The applicants propose a two-phase combination prevention intervention to address the main barriers to HIV prevention and care for transwomen (ages 18-24) in Brazil. The environments at UCSF and in Brazil are strong and the MPI team is a particular strength, with ample experience with this population in US and in-country. Using peers only in digital encounters increases the innovation of the work and its significance of the work as it may be disseminable. The premise is based on sound data of the high risk for HIV in this population. The methods are rigorous given the proposed preliminary work, pilot study embedded, and RCT at 4 clinics. The first stage work on stigma reduction at the clinics is also a strength. SBV not included. Impact could be quite high if found to be efficacious with immediate plans to scale up and good connection with Brazilian health ministry.

An application does not need to be strong in all categories to be judged likely to have major scientific impact. For example, a project that by its nature is not innovative may be essential to advance a field.

1. Significance:

Strengths

- Premise based on their recent research discovered that almost 25% of a population-based sample of young transwomen in Brazil tested positive for HIV.
- There is no HIV prevention intervention for young transwomen with evidence of efficacy outside the US.
- Their own data show young people’s HIV testing levels and care access is uneven and that almost no young transwomen regularly get tested for HIV.
- With low testing levels, it is unlikely youth are accessing HIV care.
- Intervention is well suited to the population and might be disseminable
- Could rapidly brought to scale if found to be efficacious
- Brazilian Ministry of Health is increasing prevention and care access for transwomen, including youth
- If the UH3 RCT intervention is proven efficacious, it will be scaled to all 15 ImPrEP sites across Latin America and throughout Brazil via the Brazilian Ministry of Health
- The one biomedical trial including transwomen (iPrEX) did not show efficacy for transwomen
- Pilot data show transwomen willing to use PrEP

Weaknesses

- Purely digital encounters may not be powerful enough to produce effects

2. Investigator(s):

Strengths

- The PI/PD is a Dr. P.H. with almost 20 years of experience with research with transwomen

- PI/PD has worked in Brazil, with several recent publications
- Some recent work on trans youth
- MPI Dr. Grinsztejn is an expert at testing the preliminary efficacy of HIV prevention interventions. She has been working on HIV/AIDS patient care, prevention and treatment for the last three decades.
- He is head of the STD/AIDS Clinical Research Laboratory at INI/FIOCRUZ and Director of the FIOCRUZ HIV/AIDS Clinical Trials Unit (CTU), since its establishment in 1999.
- Was the principal investigator of *Transcender*, a large Brazilian transgender women survey and has been in leadership positions in many NIAID trials groups
- Acceptable plan to share leadership and appropriate given study site in Brazil

Weaknesses

- The PI has no specific training and no prior work on adolescents
- No one is an adolescent-specific researcher
- Only 3 biosketches were included

3. Innovation:

Strengths

- Intervention will be delivered digitally by trained transwomen peers
- Interventions that require participants to interact on a particular schedule and in-person – typically used in peer navigation studies - present a number of problems for young transwomen who face substantial competing needs and barriers to participation.

Weaknesses

- ARTAS and social marketing campaign are not new (but they have good data and are adapted)

4. Approach:

Strengths

- Pilot work on a US-based cohort study of young transwomen and cross-sectional transwomen HIV risk study in Brazil
- Demonstrated ability to recruit and retain transwomen (retention over 90%)
- Intervention is private, can occur on a schedule convenient to participants, and does not require in-person interaction.
- Proven approached to recruiting and retaining transwomen in Brazil will be employed
- UG3 stage focuses on stigma reduction among providers at the 4 clinics, which is an important part of the intervention’s success
- UH3 involves 150 women to test efficacy
- Pilot data show stigma a problem
- Pilot data show clinics not youth friendly
- BeT (Shine and Transcend) intervention for young transwomen ages 18-24 years builds upon team’s prior work in Brazil and the US to implement an intervention that addresses youth-specific barriers and mitigate anti-trans stigma to increase HIV testing frequency, PrEP uptake and HIV care linkage among young transwomen ages 18-24 years old in Brazil
- Advisory group involved
- Will utilize an effective US Centers for Disease Control and Prevention (CDC) intervention
- (ARTAS) for system navigation and linkage
- Mobile implementation suits the population and will likely lead to better uptake

- Excellent go/no-go milestones that are clearly articulate and appropriate, including a pilot of the next phase
- Appropriate training of peer navigators
- Rigor supported with RCT, including pilot, and outcomes of HIV testing frequency, PrEP uptake, HIV care linkage
- Survey data included to look at mechanisms and moderators

Weaknesses

- Only 4 clinics and 150 transwomen in UH3 (which was not limited by budget, which had no upper limit)
- Intervention only 3 months long (though applicant argues they may be able to achieve a greater dose response and intervention effect in a shorter amount of time because of the possibility for a higher number of encounters)
- Shorter time-frame leaves no option to monitor frequent testing
- “Digital encounter” not described well but they don’t seem to include any phone calls, all texts and asynchronous encounters
- No justification for not offering any face –to-face encounters or voice calls
- Nothing like a standardized patient interview to test the anti-stigma attitudes of staff
- Unfortunate those under 18 are left out (but adequate justification provided)

5. Environment:

Will the scientific environment in which the work will be done contribute to the probability of success? Are the institutional support, equipment and other physical resources available to the investigators adequate for the project proposed? Will the project benefit from unique features of the scientific environment, subject populations, or collaborative arrangements?

Strengths

- The Center for Public Health Research (CPHR) at the San Francisco Department of Public Health has the resources to support the PI
- The Oswaldo Cruz Foundation Therapeutic and Prevention HIV/AIDS Clinical Trials Unit (FioTrials) is a multidisciplinary, integrated research consortium based at FIOCRUZ, one of the nation’s leading academic research institutions.
- The study has access to infrastructure available through FIOCRUZ and ongoing HIV prevention studies with the transgender community that they are implementing, including trans research experts, statisticians, and researchers.
- ImPrEP ongoing study and the Brazil’s MoH are committed to supporting the proposed study as it will generate knowledge and evidence on the effectiveness of PrEP among young transwomen

Weaknesses

- The SF site does not have any resources specific to support work in Brazil

— Responses for Protections for Minor Participants, Human Subjects, Vertebrate Animals, and
— Biohazards:
— A response for Inclusion of Women, Minorities and Children **is required from reviewers** for applications proposing Human Subjects Research

Protections for Minor Participants: No minors

Protections for Human Subjects:

Data and Safety Monitoring Plan (Applicable for Clinical Trials Only):
  - Adequate protections in place, including the consultation of a counseling psychologist.

Inclusion of Women, Minorities and Children:
- Sex/Gender:
- Race/Ethnicity:
- For NIH-Defined Phase III trials, Plans for valid design and analysis:
- Inclusion/Exclusion of Children under 18:
- All women (transwomen) 18-24 years of age, justified. Half black/African and half white.

Vertebrate Animals: na

Biohazards: N/A
□□□□□

Applications from Foreign Organizations:
- N/a

Select Agent Research:
- N/A

Resource Sharing Plans:
- appropriate

Authentication of Key Biological and/or Chemical Resources:
- N/A

Budget and Period of Support:

Recommended budget modifications or possible overlap identified:
- Small budget made possible by limited involvement of multiple, senior staff.

Additional Comments to Applicant (Optional):

CRITIQUE 3

Significance: 3
Investigator(s): 3
Innovation: 4
Approach: 5
Environment: 4

Overall Impact: Strengths of the proposal include interventions that target young transwomen in Brazil, age 18-24 years within a comprehensive strategy that aims to address stigma-related barriers to uptake of HIV testing, PreP and linkage to services through digital health (social media and SMS technology) interventions adapted to local context and specific needs of this population. The proposal draws on prior experience/interventions and significant expertise of the study team, specifically with respect to prior design of interventions and recruitment strategies for this high-risk, underserved and marginalized population. Enrollment plans appropriately target those who have not had recent access to care, as well as stakeholder/community engagement during the formative process. In addition to use of SMS/social media, the proposal includes the new application of a CDC-intervention for improvement in system navigation and linkage. The risks to participants are appropriately minimized, and the study team provides substantial training for, support of, and establishes vital referral mechanisms to address any psychological distress that may arise. Essential to the project's ability to address sustainability and scalability, analysis of cost estimates are included and appropriate. Methodology includes both appropriate qualitative and quantitative data analysis, with concrete, measurable outcomes. RCT methodology is used and appropriate to assess impact of the intervention. Finally, there are significant resources and infrastructure to support implementation and considerable research experience in both US- and Brazil-based teams with the transwomen population.

There is limited ability of this study to confirm full linkage to access ART, as project tracks linkage to HIV services, but no measure of ART uptake nor evaluation of the impact on health care workers sensitization/social marketing campaign, nor role/impact of digital navigators, on long-term retention in care and potential contribution to overall health outcomes (adherence and viral load suppression). In addition, confirmation of HIV testing, initiation of PreP and linkage to care relies on participant self-report. Finally, although required to assess the impact of the intervention, provision of cell phones and phone credit may limit the eventual sustainability/scalability of the interventions.

An application does not need to be strong in all categories to be judged likely to have major scientific impact. For example, a project that by its nature is not innovative may be essential to advance a field.

1. Significance:

Strengths

- Study proposes to work with young transwomen (18-24 years), a key population at high risk of HIV, poor HIV care outcomes and a population with limited proven interventions to successfully address primary prevention and linkage challenges
- Addresses key component of stigma-related barriers to accessing HIV diagnosis, prevention, and treatment services in this population

- Study would enhance scientific knowledge by evaluating a comprehensive strategy adapted to serve a marginalized population to increase uptake of HIV testing, access to PreP and linkage to services. Proposal draws on experience of the study team working with the transgender population and introduces the possibility of larger implementation in addition countries through pre-existing networks in 2 other Latin American countries.
- Will generate knowledge and evidence of the effectiveness of PreP among young transwomen and expand the potential benefit of PreP for this population

Weaknesses

- None

2. Investigator(s):

Strengths

- Investigators have integrated expertise, with clearly defined roles and responsibilities for both PIs and process for conflict resolution. Collaborative relationship demonstrated by work together on prior project.
- US-based PI with extensive experience in research with the transgender community and mixed methods research informing HIV behavioral surveillance research and development of interventions. Also with demonstrated research experience with PrEP demonstration project for transgender people, stigma/discrimination risk, and HIV risk/resilience work with trans-female youth. Demonstrated experience with recruitment, sampling and retaining transwomen in research and prior work in LMIC's facilitating implementation of this proposal.
- Brazil-based PI with substantial prior experience in research in multi-center research trials, in addition to acting as the PI for a demonstration project of PrEP for transgender women and a large Brazilian transgender women survey. Also serves on advisory committees for the Brazilian MOH.
- Co-investigator with demonstrated research experience and roles as technical advisor to Brazilian department of STD, AIDS and Viral hepatitis. Prior work includes role as a sub-investigator with HIV-positive women's cohort and Transgender women's cohort and coordinator of PrEP demonstration project.

Weaknesses

- None

3. Innovation:

Strengths

- Proposal introduces digital health intervention using social media and SMS technology to introduce new approach to challenges of participation and intervention effectiveness for young transwomen
- Novel intervention includes formative research component to allow adaptation to local context and adaptation to specific needs of transwomen population
- Builds upon prior published research on sampling/recruitment experience (allows refinement) to maximize enrollment of young transwomen (including targeting enrollment of youth and those with limited/no prior access to preventive HIV services.
- New application of CDC- intervention (ARTAS) for system navigation and linkage

Weaknesses

- None

4. Approach:

Strengths

- Overall strategy, methodology and analysis well reason and grounded on prior research, investigator experience, and prior use of linkage and navigation technology
- Strategies are robust; rationale for sampling and recruiting participants and formative process including targeting enrollment of those who have not had recent access to care, and the community and stakeholder engagement for developing intervention clearly outlined. Social marketing strategy outlines clear process for increasing awareness of PrEP among trans people and reduce anti-trans stigma at health facility level. Process involves formative data collection to adapt ARTAS-navigation system and pilot intervention to determine efficacy and demonstrate ability to recruit transwomen youth
- Risk to participants managed appropriately and inclusion/exclusion criteria scientifically justified. Consent process clearly defined and appropriate mechanisms in place to allow for participant withdrawal.
- Training/support for digital navigators in patient confidentiality, suicide assessment, working with LGBTQ clients, solution-focused therapy provides necessary foundation ensuring protection of study participants.
- RCT methodology appropriate to assess impact of comprehensive intervention vs SMS messaging alone; randomization to minimize selection bias.
- Project targets a high-risk population - transwomen youth age 18-24 and enrollment/recruitment process and social marketing campaign both ensure access to this population and target health care workers who will provide prevention/treatment services.
- The authors do not provide clear confirmation on how they will avoid duplicate subject enrolment, however with digital navigators assigned to individual participants as case managers and development of individual-level HIV testing, PrEP and HIV care linkage goals to guide intervention messaging and support, it is presumed that duplication can be avoided
- Timelines are appropriate, with UG3 phase centered on formative research, social marketing campaign development and implementation, and piloting of the intervention with evaluation of primary outcomes, assessment of challenges, successes and refinement of the intervention prior to implementation of UH3 phase. UH3 phase involves implementation of RCT component to evaluate overall efficacy of the intervention (uptake of HIV testing, use of PrEP, linkage to care). Proposed exit interviews via FGDs stratified by age appropriate.
- Transition milestones clearly defined, feasible and quantifiable.
- The approaches are likely to result in scalable designs, particularly given research networks in other countries and demonstrate ability to adapt technologic platforms to target hard to reach populations in a LMIC context.
- Analysis of cost estimates, cost estimates per participant and cost per HIV infection averted included and essential for evaluating sustainability and scalability.

Weaknesses

- Limited ability to confirm linkage to access ART (as project tracks linkage to HIV service provision, but no defined ability to ensure ART initiation or to evaluate impact of health care worker sensitization and social marketing campaign/social media or role of digital navigators to have impact on long-term retention in care).

- Confirmation of HIV testing, initiation of PrEP, linkage to care by self-report
- Although necessary for the intervention, provision of cell phones and phone credit may limit eventual scalability/sustainability of the intervention.

5. Environment:

Strengths

- Significant resources and research experience in both San Francisco and Rio De Janeiro, Brazil
- US-based resources include a substantial number of experienced staff to support coordination and implementation of study activities, proven capacity to conduct research with history of multiple NIH-, CDC-, HRSA- and other national & international agency funded epidemiologic, behavioral, evaluation studies of HIV, comprehensive statistical capacity for data analysis, and access to sufficient computing, secure network services, and technology support staff and resources.
- Brazil-based resources include extensive resources available through FIOCRUZ academic research institutions including direct HIV/AIDS service provision, access to ID specialists, a research environment with experience with large multi-center studies, and physical infrastructure as well as regulatory, administration, logistic, data management support.

Weaknesses

- None

— Responses for Protections for Minor Participants, Human Subjects, Vertebrate Animals, and
— Biohazards:
— A response for Inclusion of Women, Minorities and Children **is required from reviewers** for applications proposing Human Subjects Research

Protections for Minor Participants:

- Not Applicable

Protections for Human Subjects:

- All data kept on password protected network drive; study ID numbers only to identify participant information
- Participants' identities not recorded during interviews or exit focus group discussions
- Participants provide information for tracking (address, cell phone numbers, email addresses and social media site profiles); If stop participation link to study medial profiles will be disabled. However, researchers to not provide clear guideline of the limits of information to be shared on social media platforms (for example guidance to participants to not include personal identifying information on the platform itself)
- Clear and adequate plan to address psychological discomfort that may arise from questions asked during interviews and FGDs with access to psychologic support services, as well as clear support and referral pathways for participants with suicidal thoughts/attempts, history or ongoing abuse, or threat of danger to others.

Data and Safety Monitoring Plan (Applicable for Clinical Trials Only):

- Elements of data safety monitoring plan reviewed by both USCF Committee on Human Subjects research and Human subjects review at FIOCRUZ
- System to safeguard data transmission and storage elaborated– all survey data collected via password- protected tablets, storage on password-protected network services, use of software for analysis of audio recordings that requires both source data and annotation data, which are to be stored separately on secured, firewalled servers. No PII included and no access by individuals not part of the study team.

Inclusion of Women, Minorities and Children:

- Sex/Gender:
- Race/Ethnicity:
- For NIH-Defined Phase III trials, Plans for valid design and analysis:
- Inclusion/Exclusion of Children under 18:
- As the study proposes to work with young transwomen, all of the proposed study population is justified as 100% of the participants will identify as transwomen, women or otherwise not male.
- Recruitment appropriately focuses on inclusion of racial/ethnic minority or mixed-identity youth (70% target) in recognition of disproportionate impact of HIV on this population
- Exclusion of children < 18 years justified in recognition of Brazilian consent laws requiring parental/guardian written consent for inclusion in research and in recognition of marginalization of transwomen. Additional rationale provided by researchers based on US-based data that engagement in sexual risk behaviors begin at age 18 years.

Vertebrate Animals:

Biohazards:

Applications from Foreign Organizations:

- Primary institution for application is US-based, although study to be conducted in Brazil. The project does present a unique opportunity to evaluate comprehensive prevention interventions in a low-middle income context and contains a formative component to allow adaptation of the strategy in a diverse cultural context. Not only will the study potentially provide evidence for the strategy to be rolled out in other low/middle income countries, but will also provide evidence for how to adapt similar interventions and build on demonstrable successful strategies in diverse areas for underserved and marginalized young transwomen populations in the US.

Select Agent Research:

Resource Sharing Plans:

- No information on Data Sharing plan provided

Authentication of Key Biological and/or Chemical Resources:

Budget and Period of Support:

Recommended budget modifications or possible overlap identified:

Additional Comments to Applicant (Optional):

THE FOLLOWING SECTIONS WERE PREPARED BY THE SCIENTIFIC REVIEW OFFICER TO SUMMARIZE THE OUTCOME OF DISCUSSIONS OF THE REVIEW COMMITTEE, OR REVIEWERS' WRITTEN CRITIQUES, ON THE FOLLOWING ISSUES:

COMMITTEE BUDGET RECOMMENDATIONS: The budget was recommended as requested.

Footnotes for 1 UG3 HD096914-01; PI Name: Meek, Erin

NIH has modified its policy regarding the receipt of resubmissions (amended applications). See Guide Notice NOT-OD-14-074 at http://grants.nih.gov/grants/guide/notice-files/NOT-OD-14-074.html. The impact/priority score is calculated after discussion of an application by averaging the overall scores (1-9) given by all voting reviewers on the committee and multiplying by 10. The criterion scores are submitted prior to the meeting by the individual reviewers assigned to an application, and are not discussed specifically at the review meeting or calculated into the overall impact score. Some applications also receive a percentile ranking. For details on the review process, see http://grants.nih.gov/grants/peer_review_process.htm#scoring.

MEETING ROSTER

National Institute of Child Health and Human Development Special Emphasis Panel
EUNICE KENNEDY SHRIVER NATIONAL INSTITUTE OF CHILD HEALTH & HUMAN DEVELOPMENT
ZHD1 DSR-N (52)
04/09/2018

Notice of NIH Policy to All Applicants: Meeting rosters are provided for information purposes only. Applicant investigators and institutional officials must not communicate directly with study section members about an application before or after the review. Failure to observe this policy will create a serious breach of integrity in the peer review process, and may lead to actions outlined in NOT-OD-14-073 at https://grants.nih.gov/grants/guide/notice-files/NOT-OD-14-073.html and NOT-OD-15-106 at https://grants.nih.gov/grants/guide/notice-files/NOT-OD-15-106.html, including removal of the application from immediate review.

CHAIRPERSON(S)

BAUMAN, LAURIE J, PHD
PROFESSOR
DEPARTMENT OF PEDIATRICS
ALBERT EINSTEIN COLLEGE OF MEDICINE
BRONX, NY 10461

MEMBERS

DELANY-MORETLWE, SINEAD, MBBS, PHD
DIRECTOR
SCHOOL OF CLINICAL MEDICINE
UNIVERSITY OF WITWATERSRAND, JOHANNESBURG
JOHANNESBURG
SOUTH AFRICA

GUILAMO-RAMOS, VINCENT M, PHD
PROFESSOR
CENTER FOR LATINO
ADOLESCENT & FAMILY AND ADOLESCENT AIDS PROGRAM
MONTEFIORE MED CENTER
NEW YORK UNIVERSITY
NEW YORK, NY 10003

LANE, TIM, PHD
ASSOCIATE PROFESSOR
CENTER FOR AIDS PREVENTION STUDIES
UNIVERSITY OF CALIFORNIA, SAN FRANCISCO
SAN FRANCISCO, CA 94158

LUSENO, WINFRED K, PHD
RESEARCH SCIENTIST
PACIFIC INSTITUTE FOR RESEARCH AND EVALUATION
CHAPEL HILL, NC 27514

MARHEFKA, STEPHANIE LYNN, PHD
ASSOCIATE PROFESSOR
COLLEGE OF PUBLIC HEALTH
UNIVERSITY OF SOUTH FLORIDA
TAMPA, FL 33620

PALUMBO, PAUL E., MD
PROFESSOR
DEPARTMENTS OF PEDIATRICS AND MEDICINE
GEISEL SCHOOL OF MEDICINE
DARTMOUTH COLLEGE
LEBANON, NH 03756

SANDERS, RENATA A, MD
ASSISTANT PROFESSOR
DIV. OF GENERAL PEDIATRICS & ADOLESCENT MEDICINE
JOHNS HOPKINS SCHOOL OF MEDICINE
BALTIMORE, MD 21287

SANTA MARIA, DIANE M, MSN, DRPH
ASSISTANT PROFESSOR
SCHOOL OF NURSING
UNIVERSITY OF TEXAS HEALTH SCIENCE CENTER
HOUSTON, TX 77030

SIMONI, JANE, PHD
PROFESSOR
DEPARTMENT OF PSYCHOLOGY
UNIVERSITY OF WASHINGTON
SEATTLE, WA 98195

VENTUNEAC, ANA MARIA, PHD
RESEARCH SCIENTIST
ASSISTANT PROFESSOR
DEPARTMENT OF MEDICINE – INFECTIOUS DISEASES
ICAHN SCHOOL OF MEDICINE AT MOUNT SINAI
ANNENBERG BUILDING FLOOR 23 ROOM, 1468 MADISON AVE
NEW YORK, NY 10018

WECHSBERG, WENDEE M, PHD
DIRECTOR
SUBSTANCE ABUSE TREATMENT EVALUATIONS AND INTERVENTIONS
RESEARCH TRIANGLE INSTITUTE INTERNATIONAL
RESEARCH TRIANGLE PARK, NC 27709

WOLFMAN, VANESSA AUDREY, MD, MPH
HENRY M. JACKSON FOUNDATION
TECHNICAL DIRECTOR FOR PEDIATRIC AND PMTCT
CLINICAL SERVICES
6720A ROCKLEDGE DR, SUITE #400
BETHESDA, MD 20817

ZANONI, BRIAN C., MD
INSTRUCTOR
HARVARD MEDICAL SCHOOL
MASSACHUSETTS GENERAL HOSPITAL
55 FRUIT STREET
BOSTON, MA 02114

SCIENTIFIC REVIEW OFFICER

HOUSTON, KIMBERLY LYNETTE, MD
SCIENTIFIC REVIEW OFFICER
SCIENTIFIC REVIEW BRANCH (SRB)
EUNICE KENNEDY SHRIVER NATIONAL INSTITUTE
OF CHILD HEALTH AND HUMAN DEVELOPMENT
BETHESDA, MD 20892

EXTRAMURAL SUPPORT ASSISTANT

EDWARDS, MAURICE L.
EXTRAMURAL SUPPORT ASSISTANT
SCIENTIFIC REVIEW BRANCH, DER
EUNICE KENNEDY SHRIVER NATIONAL INSTITUTE OF
CHILD HEALTH AND HUMAN DEVELOPMENT, NIH, DHHS
BETHESDA, MD 20817

Consultants are required to absent themselves from the room during the review of any application if their presence would constitute or appear to constitute a conflict of interest.
